# Behavioural inventory of the giraffe (*Giraffa camelopardalis*)

**DOI:** 10.1186/1756-0500-5-650

**Published:** 2012-11-22

**Authors:** Peter A Seeber, Isabelle Ciofolo, André Ganswindt

**Affiliations:** 1 Department of Production Animal Studies, Faculty of Veterinary Science, University of Pretoria, Private Bag X04, Onderstepoort 0110, South Africa; 2 Le Pré Commun, Aspet 31160, France; 3 Department of Zoology and Entomology, Mammal Research Institute, University of Pretoria, Pretoria 0002, South Africa

**Keywords:** Giraffa camelopardalis, Ethogram, Behavioural activity, Abnormal repetitive behaviour, Social interaction, Hwange National Park, Entabeni Game Reserve, National Zoological Gardens of South Africa

## Abstract

**Background:**

Numerous factors like continuous habitat reduction or fragmentation for free-ranging giraffes (*Giraffa camelopardalis*) as well as e.g. suboptimal housing conditions for animals in captivity might lead to behavioural alterations as part of the overall adaptation process to the changing living conditions. In order to facilitate current and future studies on giraffe behaviour, a comprehensive ethogram was compiled based on existing literature, as well as observations on giraffes in the wild (Hwange National Park, Zimbabwe; Entabeni Game Reserve, South Africa), and in captivity (National Zoological Gardens of South Africa, Pretoria).

**Findings:**

The resulting ethogram lists 65 different behavioural patterns, which were described and grouped into seven categories: General activities, Abnormal repetitive behaviours, General interactions, Bull-Cow behaviour, Bull-Bull behaviour, Cow-Bull behaviour, Maternal behaviours, and Interactions by calves. The behaviours were further described regarding a presumed purpose, particularly with respect to social interactions and sexual behaviour. Contradictory descriptions from previous studies were considered and discussed in comparison with our own observations.

**Conclusions:**

This ethogram provides a basis for current and future studies by suggesting a terminology which can be used for harmonizing behavioural observations, thus helping to facilitate comparability of future results. Subsequently, a better understanding of the behavioural ecology of giraffes in the wild as well as in captivity could aid future conservation efforts.

## Background

The giraffe (*Giraffa camelopardalis*) is the tallest land-living animal and the only extant species of its genus [1]. Although there is still uncertainty about the exact number and distribution of subspecies within *Giraffa*, a division into nine subspecies are generally accepted [2]. Two of these subspecies are currently listed as endangered by the International Union for Conservation of Nature and Natural Resources [3]. The giraffe naturally inhabits a variety of habitats, from deserts to more heavily vegetated bush- and tree savannah [4,5], and there is evidence for habitat-related adjustments of occurring social structures and of particular behaviours, such as stable social structures and dominance hierarchies instead of fission-fusion structures [6].

Over the past couple of decades, the number of giraffes has declined considerably across Africa, presumably due to direct and indirect anthropogenic impact, such as extensive poaching, habitat destruction, and rinderpest [7-9]. As a consequence, several of today’s giraffe populations are isolated and live in detached habitat fragments or fenced reserves [7,8]. By restricting the natural tendency of giraffes to roam vast areas in search for conspecifics, further implications in terms of behavioural alterations are easily conceivable [6,10,11]. These alterations might even have long-term effects on e.g. intraspecific competition, predator-prey relationships, or parasite transmission amongst other factors [6,10,11], and might have to be considered in future conservation plans for affected populations. However, data on the giraffe’s ethology and its variation should be available in order to provide information for an integrated conservation approach [12].

Monitoring of wildlife behaviour is also a valuable and frequently used tool to provide information about the health and welfare status of animals in captivity [13,14]. However, the interpretation of behavioural data is not robust, and relies heavily on preliminary defined criteria [15]. In order to obtain reliable results, the respective behavioural patterns must be adequately defined [16]. To allow cross-institutional comparison of collected data and to contribute to a reliable base of information, behaviour must be measured in a distinct and standardised way. Thus, the use of an accurate established ethogram is highly recommendable, not least because it helps to prevent ‘drift’ during the course of observation and also in order to facilitate methodology and results [17].

In terms of available behavioural data for the giraffe, many of the contributing studies only cover specific behavioural classes and at times, these studies use inconsistent terminology or innovate purpose-built definitions for certain behaviours e.g. [18-23]. Hence, data to build upon is rather limited. In this paper, we therefore aim to provide a full descriptive catalogue of the giraffe’s behavioural repertoire for observations of wild and captive animals. The compiled ethogram is structured into several categories, which are, in the case of intraspecific interactions, subdivided by sex and roughly predefined age classes of the acting individuals, as well as the animals the behaviour is presumably directed to. The definitions and general remarks provided will hopefully be of practical value in terms of producing more comparable ethological data in the future.

Observations were conducted at three different study sites: Hwange National Park (HNP), Zimbabwe, Entabeni Game Reserve (EGR), South Africa, and at the National Zoological Gardens of South Africa (NZG) in Pretoria.

## Findings

### Methods

#### General method

In a similar approach to other studies e.g. [24,25], the behaviours reported in this paper were compiled from several sources. In order to assess as many of the behaviours shown by giraffes as possible, numerous peer-reviewed articles, dissertations and theses, and other publications (Appendix), focussing on descriptions of giraffe behaviour or at least partly addressing the topic, were reviewed for behavioural descriptions and definitions e.g. [2,4,5,9,16,18-23,26-50]. In addition, observations were conducted in three different environments, in order to confirm, refine, and if necessary extend existing descriptions of giraffe behaviour. In case of future observations though, variations in methodology and flexibility of the research has to be noted.

All behaviours were described as brief and definite as possible, according to the descriptions by other authors and our own observations. In this regard, we tried to comment regarding the behaviours apparent social and/or ecological context, and assumed purpose. The resulting list of behaviours is divided into two main groups; *Activities* (characterised by the absence of any social context) and *Interactions* (characterised by the presence of some kind of interaction between animals).

#### Literature review

In total, 104 publications (93 scientific articles, 2 books, 3 PhD theses, 2 MSc dissertations and 4 other publications) on giraffe behaviour, ecology, and general biology were reviewed for descriptions of behavioural patterns in wild and captive giraffes, listed in the Appendix.

#### Original observations

Giraffes were predominantly observed using *ad-libitum* and *all-occurrence* sampling [51]. As an example of a near-natural environment, wild giraffes were observed in HNP for thirteen weeks, between November and December 2010, and from March to April in 2011. During a total observation time of 272 hours, 1264 sightings were recorded (345 sightings of mature males, 752 of mature females, 159 of juveniles / subadult individuals). HNP covers 14.650 km^2^ in western Zimbabwe and is entirely unfenced. A presumably stable population of roughly 2800 giraffes are estimated to live in HNP and adjacent areas [3]. Lions as the giraffe’s main predators are abundant [52], and also other large predators such as spotted hyenas, cheetahs and leopards are present. Observations were conducted at several water holes and open plains in the Main Camp area, ranging from Guvalala Pan to Ngweshla Pan.

About 40 giraffes were additionally observed on a daily basis in EGR for three weeks in September 2011. EGR is a private game reserve, covering 250 km^2^ and is entirely fenced. An otherwise unmanaged population of about 45 giraffes were kept in the reserve during the time of observation. Lions, cheetahs, and leopards are also present.

In order to collect data on an abnormal repetitive behaviour in a captive animal, two adult giraffes (1 male, 1 female) housed at NZG, were also briefly observed for 7 hours in February 2011.

## Results and Discussion

A total of 65 different behaviours could be identified. These behaviours were subdivided into 30 *Activities* and 35 *Interactions*. Activities were subdivided further into *General activities* (Additional file 1: Table S1) and *Abnormal repetitive behaviours* (Additional file 2: Table S2). Interactions were structured by sex and age class of the acting animal, and of the animals the behaviour is presumably directed to. This resulted in *General interactions* (Additional file 3: Table S3), *Bull-Cow behaviour* (Additional file 4: Table S4), *Bull-Bull behaviour* (Additional file 5: Table S5), *Cow-Bull behaviour* (Additional file 6: Table S6), *interactions by calves* (Additional file 7: Table S7), and *maternal behaviours* (Additional file 8: Table S8). No behaviours were found being performed exclusively between cows.

### Activities

As mentioned above, behaviours allocated to the category *Activities* are not related to any type of interactive behaviour and also not restricted to one sex or age class. Behaviours of the *Activities* category were further subdivided into *General Activities* (Additional file 1: Table S1) and *Abnormal repetitive behaviours* (Additional file 2: Table S2).

### Abnormal repetitive behaviours

As in other species, it is assumed that abnormal repetitive behaviours often develop in captive animals due to a time budget shift in the daily activity pattern [46,47]. Giraffes in captivity spend considerably less time feeding compared to the amount of time giraffes *browse* in the wild [16,20].

### Interactions

This section includes behaviours which are characterised by any type of direct or indirect social interaction between individual giraffes. Behaviours of the *Interactions* category were further subdivided into *General Interactions* (Additional file 3: Table 3), *Bull - Cow Behaviour* (Additional file 4: Table S4), *Bull - Bull Behaviour* (Additional file 5: Table S5), Cow - Bull *Behaviour* (Additional file 6: Table S6), b*ehavioural Interactions by Calves* (Additional file 7: Table S7) and *maternal behaviours* (Additional file 8: Table S8). All behaviours performed between cows (*cow-cow*) were also observed between other constellations of sex and age, thus listed under *general interactions*.

This ethogram was compiled to serve as a basis for current and future studies designed to further examine the complex behavioural patterns of the species. Based on our own observations, several often older descriptions could be verified and even new insights added to what is stated in literature.

The classification of the described behaviours into *activities* and *interactions* might appear rather clear from a definition point of view, but should be used with precaution, because the complete intention and purpose of an observed behaviour always remains an interpretation based on a projection of the observer’s conception. The animal’s behaviour can not be reduced to the sum of different behavioural acts, which is why clear and precise terminology is essential to create a common language understandable among human observers and to contribute to the understanding of wildlife behaviour.

Regarding social interactions not restricted to one sex or age class (*General Interactions*), it is worth noting that many of these behaviours were originally described as exclusively exaggerated by one sex, or by a specific age class. However, during our observations, we also register the performance of these behaviours by the respective opposite sex, or across age classes, respectively.

The section on play behaviour was kept rather short and comprehensive. For the sake of brevity, all behaviours of the same obvious (play) intention were summarised. Nevertheless, future studies might be able to reveal various forms of play behaviour in giraffe, similar to that of other ungulates, although probably not as pronounced as e.g. in horses [24].

Several behaviours, although often only observed in form of an attempt (e.g. mounting, mating, nursing) are classified as separate behaviours in this ethogram, because attempts seem to be distinct and important, therefore these behaviours might be considered as a separate sub-section in an ethogram used for observations.

It must be also mentioned, that for the visual communication of dominance, contradictory descriptions are given in literature. Pratt and Anderson [5] report that a dominant bull will walk towards an opponent with its head held high, intending to look as big as possible. On the contrary, Dagg [9] states that a dominant bull, threatening an opponent will carry his head deep with the neck parallel to the ground, as if assuming a fighting position. We suggest that both observations are adequate and that communication of dominance might vary with the distance between opponents. In this regard, the “head-high” posture could be assumed for a distance of more then two body lengths, while the “fight” posture would be assumed with the opponent in close proximity, as it has been seen during our own observations. The typical intention of a threatening giraffe bull is often expressed by an arched and tensed neck (see *Dominance gesture*), as it is also seen in other ungulates, e.g. horses [53] or reindeer [54]. The visual communication of submission is contrary to that of *dominance* and thus is also described contradictory in literature. According to Pratt and Anderson [5,27], the subdominant individual will carry its head low to look smaller than it is, in order to not provoke aggression. Dagg [9] reports that inferior giraffe bulls stand with an erect neck and the nose pointed upwards, assuming a feeding position and thereby exposing the body to attacks. As well as for *dominance*, a distance dependent expression for submission might be considered. In this regard, the plasticity of social behaviour and communication patterns should be borne in mind during conduction and interpretation of behavioural observations.

## Conclusions

Observed behaviours should be interpreted carefully and the researcher should consider the animal’s intention not only for the moment and place of observation but also on a larger temporal and spatial scale. An animal’s original intention is in many cases difficult to evaluate and rather oblique, which applies particularly to large animals like the giraffe with its rarely assessable visual facilities [26]. Furthermore, olfactory cues and insufficient recognition regarding long distance communication via infrasound make it sometimes difficult to unequivocally relate a certain observed behavioural event to a specific category (von Muggenthaler, Baes, Hill, Fulk, Lee, unpublished results), therefore the division of interactions and activities not related to a social context remain somewhat arbitrary.

A comprehensive and reliable tool to monitor giraffe behaviour in the wild as well as in captivity is a necessity to gain a better understanding of the giraffe’s life-history requirements. Subsequently, gaining a better understanding of giraffe behaviour will help to develop more effective conservation strategies for improving giraffe management in the wild and in captivity by creating species-tailored management plans.

## Appendix

### Sources used for the compilation of the giraffe ethogram

Awange JL, Aseto O, Ong’ang’a O (2004): A case study on the impact of giraffes in Ruma National Park, Kenya. *Journal of Wildlife Rehabilitation* 27, 16-21.

Backhaus D (1961): Beobachtungen an Giraffen in Zoologischen Gärten und freier Wildbahn. *Instit. Pares. Nat. Cong. Ruanda-Urund*, Bruxelles. 202 pp.

Bashaw MJ, Bloomsmith MA, Maple TL, Bercovitch FB (2007): The structure of social relationships among captive female giraffe (*Giraffa camelopardalis*). *Journal of Comparative Psychology* 121 No.1, 46-53.

Bashaw M (2010): Consistency of captive giraffe behaviour under two different management regimes. *Zoo Biology* 29, 1-8.

Baxter E & Plowman AB (2001): The effect of increasing fibre on feeding, rumination and oral stereotypies in captive giraffes *(Giraffa camelopardalis). Animal Welfare* 10, 281-290.

Bercovitch FB, Bashaw MJ, Penny CG, Rieches RG (2004): Maternal investment in captive giraffes. *Journal of Mammalogy* 85 No. 3, 428-431.

Bercovitch FB, Bashaw MJ, del Castillo SM (2006): Sociosexual behaviour, male mating tactics, and the reproductive cycle of giraffe Giraffa camelopardalis*. Hormones and Behavior* 50, 314-321.

Bercovitch FB & Berry PS (2009): Ecological determinants of her size in the Thornicroft’s giraffe in Zambia. *African Journal of Ecology* 48, 962-971.

Bercovitch FB & Berry PS (2009): Reproductive life history of Thornicroft‘s giraffe in Zambia. *African Journal of Ecology* 48, 535-538.

Bernhard A, Eulenberger K (2003): Hand-rearing of a giraffe (*Giraffa camelopüardalis*) at Leipzig zoo. *Erkrankungen der Zootiere* 41, 327-328.

Berry PS (1978): Range movements of giraffe in the Luangwa Valley, Zambia. *East African Wildlife Journal* 16, 77-83.

Bredin IP, Skinner JD, Mitchell G (2008): Can osteophagy provide giraffes with phosphorus and calcium? *Onderstepoort Journal of Veterinary Research* 75, 1-9.

Brenneman RA, Louis EEJr, Fennessy J (2009): Genetic structure of two populations of the Namibian Giraffe, *Giraffa camelopardalis angolensis*. *African Journal of Ecology* 47, 720-728.

Brenneman RA, Bagine, RK, Brown, DM, Ndetei R, Louis EEJr (2009): Implications of closed ecosystem conservation management: the decline of Rothschild’s giraffe (*Giraffa camelopardalis rothschildi*) in Lake Nakuru National Park, Kenya. *African Journal of Ecology* 47, 711-719.

Blomqvist PA & Renberg L (2007): Feeding behaviour of Giraffe (*Giraffa camelopardalis*) in Mokolodi Reserve, Botswana. *University of Uppsala, Minor Fied Study.*

Bourliere F (1961): The sex ratio of the giraffe. *Mammalia* 25, 467-471.

Brown DM, Brenneman RA, Koepfli KP, Pollinger JP, Milá B, Georgiadis NJ, Louis EEJr, Grether GF, Jacobs DK, Wayne RK (2007): Extensive population structure in the giraffe. *BMC Biology.* doi:10.1186/1741-7007-5-57.

Caister LE, Shields WM, Gosser A (2003): Female tannin avoidance: a possible explanation for habitat and dietary segregation of giraffes (*Giraffa camelopardalis peralta*) in Niger. *African Jorunal of Ecology* 41, 201-210.

Cameron EZ & du Toit J (2007): Winning by a neck: tall giraffes avoid competing with shorter browsers. *American Naturalist* 169, 130-135.

Cameron EZ & du Toit J. (2005): Social influence on vigilance behaviour in giraffes, *Giraffa camelopardalis*. *Animal Behaviour* 69, 1337-1344.

Coe MJ (1967): Necking behaviour in the Giraffe. *Journal of Zoology, London* 151, 313-321.

Ciofolo I, Ambouta K, Le Pendu Y (2009): Les dernières girafes d’Afrique de l’ouest: sauvegarde assure ou avenbir menacé? *Rev. Écol. (Terre Vie)* 64, 351-358.

Ciofolo I & Le Pendu Y (2002): The feeding behaviour of giraffe in Niger. *Mammalia* 66, 183-194.

Ciofolo I (1995): West Africa’s last giraffes: the conflict between development and conservation. *Journal of Tropical Ecology* 11, 577-588.

Claus M, Franz-Odendaal TA, Brasch J, Castell JC, Kaiser T (2007): Tooth wear in captive giraffes (*Giraffa camelopardalis*): Mesowear analysis classifies free-ranging specimens as browsers but captive ones as grazers. *Journal of Zoo and Wildlife Medicine* 38, 433-445.

Clauss M, Flach EJ, Lechner-Doll M, Hatt JM (2003) Reaction of a group of captive giraffe *(Giraffa camelopardalis*) to the introduction of a tannin-containing pellet. *Erkrankungen der Zootiere* 41, 343.

Dagg, AI (1962) The distribution of the giraffe in Africa. *Mammalia* 26, 497-505.

Dagg AI (1970): Tactile encounters in a Herd of Captive Giraffes. *Journal of Mammalogy* 51 No. 2, 279-287.

Dagg AI (1971): Giraffa camelopardalis. *Mammalian species* 5, 1-8.

Dagg AI & Taub A (1970): Flehmen. *Mammalia* 34 No. 4, 686-695.

del Castillo SM, Bashaw MJ, Patton ML, Rieches RR, Bercovitch FB (2005): Fecal steroid analysis of female giraffe (*Giraffa camelopardalis*) reproductive condition and the impact of endocrine status on daily time budgets. *General and Comparative Endocrinology* 141, 271-281.

Dumonceaus GA, Baumann JE, Camilo GR (2006): Evaluation of progesterone in feces of captive reticulated giraffe (*Giraffa camelopardalis reticulate*). *Journal of Zoo and Wildlife Medicine* 3,255-261.

Du Toit JT & Yetman CA (2005): Effects of body size on the diurnal activity budget of African browsing ruminants. *Oecologia* 143 No. 2, 317-325.

Fennessy J (2004): Ecology of desert-dwelling giraffe *Giraffa camelopardalis angolensis* in northwestern Namibia. *University of Sydney, Australia, Phd thesis.*

Fennessy J (2009): Home range and seasonal movements of *Giraffa camelopardalis angolensis* in the Northern Namib Desert. *African Journal of Ecology* 47 No. 3, 318-327.

Fennessy J & Brown D (2010): *Giraffa camelopardalis. IUCN 2010. IUCN Red List of Threatened Species.* Version 2010.3.

Fleming PA, Hofmeyr SD, Nicolson SW, du Toit JT (2006): Are giraffes pollinators of flower predators of Acacia nigrescens in Kruger National Park, South Africa? *Journal of Tropical Ecology* 22, 247-253.

Foster J & Dagg I (1972): Notes on the biology of the giraffe. *East African Wildlife Journal* 10, 1-16.

Foster JB (1966) The giraffe of Nairobi National Park: home ranges, sex ratios, the herd and food. *East African Wildlife Journal* 4, 139-148

Gilbert DE, Loskutoff NM, Dorn CG, Nemec LA, Calle PP, Kraemer DC, Threlfall WR, Raphael BL (1988): Hormonal manipulation and ultrsonographic monitoring of ovarian activity in the giraffe. *Theriogenology* 29, 248.

Ginnett TF, Demment MW (1997) Sex differences in giraffe foraging behaviour at two spatial scales. *Oecologia* 110, 291-300.

Ginnett TF, Demment MW (1999): Sexual segregation by Masai giraffes at two spatial scales. *African Journal of Ecology* 37, 93-106.

Giraffe Conservation Foundation: The Facts. URL: http://www.giraffeconservation.org/giraffe_facts.php?pgid=40 (accessed January 20, 2011).

Gombe S, Kayanja FI (1974): Ovarian progestins in Masai giraffe (*Giraffa camelopardalis*). *Journal of Reproduction and Fertility* 40, 45-50.

Goodman PS, Tomkinson AJ (1987): The past distribution of giraffe in Zululand and its implications for reserve management. *South African Journal of Wildlife Research* 17, 28-23.

Grubb P (2005): Artiodactyla. In: D. E. Wilson and D. M. Reeder (eds), Mammal Species of the World. A Taxonomic and Geographic Reference (3rd ed), 637-722. Johns Hopkins University Press, Baltimore, USA.

Hall-Martin AJ (1974): Notes on utilization of different vegetation types by giraffe. *South African Journal of Science* 70, No. 4, 122-123.

Hall-Martin AJ (1975): Studies on the biology and productivity of the giraffe, *Giraffa camelopardalis*. *D.Sc. thesis*, University of Pretoria.

Hall-Martin AJ, Skinner JD, van Dyk JM (1975): Reproduction in the giraffe in relation to some environmental factors. *East African Wildlife Journal 13, 237-248.*

Hall-Martin AJ, Skinner JD, Hopkins BJ (1978): The development of the reproductive organs of the male giraffe, *Giraffa camelopardalis*. *Journal of Reproduction and Fertility* 52, 1-7.

Hall-Martin AJ & Skinner JD (1978): Observations on puberty and pregnancy in female giraffe (*Giraffa camelopardalis*). *South African Journal on Wildlife Research* 8, 91-94.

Hassanin A, Ropiquet A, Gourmand AL, Chardonnet B, Rigoulet J (2007): Mitochondrial DNA variablity in Giraffa camelopardalis: consequences for taxonmoy, phlyogeography and conservation of giraffes in West and central Africa. *C.R. Biologies* 330, 265-274.

Horwich R, Ktichen C, Wangel M, Ruthe R (1983): Behavioral development in Okapis and Giraffes. *Zoo Biology* 2, 105-125.

Innis AC (1958): The behaviour of giraffe, *Giraffa camelopardalis*, in the eastern Transvaal*. Proceedings of the Zoological Society, London* 131, 245-275.

Isobe N, Nakao T, Shimada M, Fukumoto Y, Watanabe H, Minami S, Noda A, Yoshimura Y (2007) Fecal progestagen and estrone during pregnancy in a giraffe: a case report. *Journal of Reproduction and Development* 53, 159-164.

Jolly L (2003): Giraffe husbandry manual. URL: http://www.aszk.org.au/docs/giraffe.pdf (*accessed September 29 2010*).

Kok, OB, Opperman, DP (1980): Feeding behaviour of giraffe *Giraffa camelopardalis* in the Willem-Pretorius-Game-Reserve, Orange Free State. *South African Journal of Wildlife Research* 10, 45-55.

Kristal MB, Noonan M (1979): Note on sleep in captive giraffes (*Giraffa camelopardalis reticulata*). *South African Journal of Zoology* 14, 108.

Kristal MB, Noonan M (1979): Perinatal maternal and neonatal behaviour in the captive reticulated giraffe*. South African Journal of Zoology* 14, 103-107.

Kruger JW (1994): The feeding ecology and behaviour of re-introduced giraffe (*Giraffa camelopardalis*) in the Kalahari Gemsbok National Park. Msc Thesis, University of Pretoria.

Lamprey HF (1963): Ecological separation of the large mamal species in the Tarangire game reserve, Tanganyika. *African Journal of Ecology* 1, 63-92.

Langman VA (1978): Giraffe pica behaviour and pathology as indicator of nutritional stress. *The Journal of Wildlife Management* 42, 141-147.

Leuthold BM & Leuthold W (1972) Food habbits of giraffe in Tsavo National Park, Kenya. *East African Wildlife Journal* 10, 129-141.

Leuthold BM & Leuthold W (1978) Daytime activity patterns of gerenuk and giraffe in Tsavo National Park, Kenya. *East African Wildlife Journal* 16, 231-243.

Leuthold B (1979): Social organization and behaviour of giraffe in Tsavo East National Park. *African Journal of Ecology* 17, 19-34.

Leuthold BM & Leuthold W (1978): Ecology of giraffes in Tsavo-East National Park, Kenya. *East African Wildlife Journal* 16 No. 1, 1-20.

Le Pendu Y, Ciofolo I, Gosser A (2000): The social organization of giraffes in Niger. *African Journal of Ecology* 38,78-85.

Le Pendu Y & Ciofolo I (1999): Seasonal movements of giraffes in Niger. *Journal of Tropical Ecology* 15, 341-353.

Leroy R, de Visscher Ma, Halidou O, Boureima A (2009): The las African white giraffes live in farmer’s fields. *Biodiversity and Conservation* 18, 2663-2677.

Loskutoff NM, Walker L, Ott-Joslin JE, Raphael BL, Lasley BL (1986): Urinary steroid evaluations to monitor ovarian function in exotic ungulates: II. Comparison between the giraffe (*Giraffa camelopardalis*) and the Okapi (*Okapia johnstoni*). *Zoo Biology* 5, 331-338.

Lueders I, Niemuller C, Pootoolal J, Rich P, Gray C, Streich WJ, Hildebrandt TB (2009): Sonomorphology of the reproductive tract in male and pregnant and nonpregnant female Rothschild‘s giraffes (*Giraffa camelopardalis rothschildi*). *Theriogenology* 72, 22-31.

Lueders I, Hildebrandt TB, Pootoolal J, Rich P, Gray C (2009): Ovarian ultrasonography correlated with fecal progestins and estradiol during the estrous cycle and early pregnancy in giraffes (*Giraffa camelopardalis rothschildi*). *Biology of Reproduction* 81, 989-995.

Lydekker R (1904): On the subspecies of *Giraffa camelopardalis*. *Proceedings of the Zoological Society of London* 1, 202-207.

Martinez del Castillo G (2006): Correction of the hoofs due to abnormal growth in juvenile captive giraffes. *Redvet* 7, 2-6.

Mitchell G & Skinner JD (2003): On the origin, evolution and phylogeny of giraffes *Giraffa camelopardalis. Transactions of the Society of South Africa* 58 No. 1, 51-73.

Mitchell G, van Sittert SJ, Skinner JD (2009): Sexual selection is not the origin of long necks in giraffes. *Journal of Zoology* 278, 281-286.

Nowak RM (1999): Walker's Mammals of the World. Johns Hopkins University Press.

Nesbit Evans EM (1970) The reaction of a group of Rothschild’s giraffe to a new environment. *East African Wildlife Journal* 8, 53-62.

Parker DM (2004): The feeding biology and potential impact of introduced giraffe (*Giraffa camelopardalis*) in the Eastern Cape Province, South Africa. MSc thesis, Rhodes University.

Patten RA (1940): Breeding the giraffe (*Giraffa camelopardalis*). *Australian Zoologist* 9, 452-454.

Pellew RA (1983): The giraffe and its food resource in the Serengeti. I: Composition, biomass and production of available browse. *African Journal of Ecology* 21, 241-267.

Pellew RA (1983): The giraffe and its food resource in the Serengeti. II. Response of the giraffe population to changes in the food supply. *African Journal of Ecology* 21, 269-283.

Pellew RA (1984): Food consumption and energy budgets of the giraffe. *Journal of Applied Ecology* 21, No. 1, 141-159.

Périquet S, Valeix M, Loveridge AJ, Madzikanda H, MacDonald DW & Fritz H (2010): Individual vigilance of African herbivores while drinking: the role of immediate predation risk and context. *Animal Behaviour* 79, 665-671.

Phillips K (2006): How giraffes keep the pressure up. *Journal of experimental Biology* 209, iii.

Pournele GH (1955): Notes on the reproduction of a Baringo giraffe. *Journal of Mammalogy* 4, 574.

Pratt DM & Anderson VH (1982): Population, distribution and behaviour of giraffe in the Arusha National Park, Tanzania. *Journal of Natural History* 16, 481-489.

Pratt DM & Anderson VH (1985): Giraffe social behaviour. *Journal of Natural History* 19, 771-781.

Pratt DM & Anderson VH (1979): Giraffe cow-calf relationships and social development of the calf in the Serengeti. *Zeitschrift für Tierpsychologie* 51, 233-251.

Sauer JJ, Theron GK, Skinner JD (1977): Food preferences of giraffe *Giraffa camelopardalis* in the arid bushveld of the eastern Transvaal. *South African Journal of Wildlife Research* 7, 53-59.

Shorrocks B & Croft DP (2009): Necks and networks: a preliminary study of population structure in the reticulated giraffe (*Giraffa camelopardalis reticulate* de Winston). *African Journal of Ecology* 47, 374-381.

Seeber PA, Ndlovu HT, Duncan P, Ganswindt A (2012a): Grazing behaviour of the giraffe (*Giraffa camelopardalis*) in Hwange National Park, Zimbabwe. *African Journal of Ecology*, DOI: 10.1111/j.1365-2028.2011.01314.x

Suraud JP (2011): Identifying conservation constraints for the last West African giraffe: population dynamics determining factors and spatial distribution pattern. PhD thesis, University of Lyon.

Tarou Fernendez L, Bashaw MJ, Sartor RL, Bouwens NR, Maki TS (2008): Tongue twisters: feeding enrichment to reduce oral stereotypy in giraffe. *Zoo Biology* 27, 200-212.

Tarou L, Bashaw MJ, Maple TL (2000): Social attachment in giraffe: Response to social separation. *Zoo Biology* 19, 41-51.

van der Jeugd HP & Prins HT (2000): Movements and group structure of giraffe (*Giraffa camelopardalis*) in Lake Manyara National Park, Tanzania. *Journal of Zoology* 251, 15-21.

Veasey JS, Waran NK, Young RJ (1996): On comparing the behaviour of zoo housed animals with wild conspecifics as a welfare indicator, using the giraffe (*Giraffa camelopardalis*) as a model. *Animal Welfare* 5, 139-153.

von Muggenthaler E, Baes C, Fulk R, Lee A (1999): Infrasound and low frequency vocalizations from the giraffe; Helmholtz resonance in biology. *Proceedings of Riverbanks Consortium.*

Wang T, Brondum E, Hasenkam M, Secher N, Bertelsen M, Grondahl C, Kastberg K, Buhl R, Aalkjaer C, Baandrup U, Nygaard H, Smerup M, Sloth E, Nissen P, Runge M (2008): Blood flows and pressures when the giraffe lowers its head. *Comparative Biochemistry and Physiology* 3, 107-108.

Western D (1971): Giraffe chewing a Grant’s gazelle carcass. *East African Wildlife Journal* 9, 156-157.

Woods TD (1972): The precopulatory behaviour in male giraffe. *Lammergeyer* 17, 67.

Wyatt JR (1971): Osteophagia in Masai giraffe. *East African Wildlife Journal* 9, 157.

Young TP & Isbell LA (1991): Sex differences in Giraffe feeding ecology: energetic and social constraints. *Ethology* 87, 79-89.

Yuan J, Dong G, Zhang D (2004): The activity pattern of giraffe (*Giraffa camelopardalis*) during winter. *Chinese Journal of Zoology* 39, 76-78.

## Competing interests

The authors declare that they have no competing interests.

## Authors’ contributions

PAS and AG conceived the study and drafted the manuscript. IC reviewed the initial draft and contributed on information and behavioural interpretation. All authors contributed to, read, and approved the final manuscript.

## Supplementary Material

Additional file 1: Table S1 General Activities [2,4,9,16,20,21,23,26-28],[31-35,38-52,55-62]. Click here for file

Additional file 2: Table S2 Abnormal repetitive behaviours [16,20,26,30,45,46]. Click here for file

Additional file 3: Table S3 General Interactions [5,18,19,23,27,42,44,47-50],[59]. Click here for file

Additional file 4: Table S4 Bull Cow Behaviour [19,23,27]. Click here for file

Additional file 5: Table S5 Bull-Bull Behaviour [9,18,23,41,60]. Click here for file

Additional file 6: Table S6 Cow - Bull Behaviour [23,27]. Click here for file

Additional file 7: Table S7 Behavioural Interactions by Calves [5,42,47]. Click here for file

Additional file 8: Table S8 Maternal behaviour [41,42,55]. Click here for file
