# Supplementary material for: Behavioural inventory of the giraffe (Giraffa camelopardalis)
Source: BMC Res Notes. 2012 Nov 22;5:650. doi: 10.1186/1756-0500-5-650 (PMC3599642; doi:10.1186/1756-0500-5-650)
Supplement: Additional file 1: Table S1 — General Activities [2,4,9,16,20,21,23,26-28],[31-35,38-52,55-62]. [file 1756-0500-5-650-S1.doc]

**Table 1** General Activities

| ***browse*** | The giraffe uses tongue and/or lips to gather and ingest browse from trees or shrubs. Shoots, leaves, or leaflets are stripped off or picked from the plant. In free-ranging giraffe, a large part of the daily time budget is occupied by browsing (about 12 to 16 hours [21, 28, 29]). The time spent chewing between bites is usually used to *scan* [21]. Browsing is to distinguish from *feeding* and *grazing*. | | | | |
| --- | --- | --- | --- | --- | --- |
| ***feed*** (in captive animals) | The giraffe ingests food other than browse, such as concentrates or hay; captive animals are often fed on hay and concentrates from cribs or elevated feed buckets [20]. On the contrary to browsing, giraffe spend considerably less time feeding on concentrates due to a higher nutrient content and easier accessibility; while giraffe in the wild spend up to 16 hours browsing, captive individuals only feed as little as 4-6 hours [16, 29, 30]. | | | | |
| ***drink***  In order to drink from a water source on or below ground level, the giraffe has to splay its forelegs out laterally, and additionally flex its carpal joints to reach the water surface. Water is swallowed in this position as well. One drinking bout, on average, lasts for about 17 seconds (own observation); in the wild, drinking is often preceded by long periods of scanning and pacing along the banks of the water body; drinking is often interrupted to shake birds off or to merely scan, raising the head up but remaining with splayed out legs (own observation). | | | |  | |
| ***drink (from trough)***  Animals in captivity are usually provided an elevated water source, making the typical drinking position obsolete [16]. No data on drinking time and count of bouts was available. | | | | | |
| ***ruminate***  Rumination is chewing a bolus of already ingested food after regurgitation. In the giraffe, regurgitation of a bolus is usually visible on the outside of the throat and therefore the subsequent chewing movement that is not proximately following prior food intake is considered rumination; the giraffe can ruminate while standing, lying, or even while walking [29, 31]. | | | | | |
| ***scan***  The individual actively observes its surroundings. Vigilance is most obvious when the animal is standing still with an erect neck and appears to be actively watching, but scanning is apparently done synchronously with many other behaviours (e.g. while ruminating, walking, or between feeding bouts) [2, 32]. The giraffe’s visual abilities are assumed to be its most important feature of predator defence [32]. Backhaus [33, quoted 9] has proven high developed visual abilities in giraffes. | | | | |  |
| ***walk***  The animal moves in a four-beat locomotion which is distinct from the three-beat *cantering* also due to its lack of a phase of suspension. In walk, there is a tendency to pacing, as both legs on one side might swing forward almost contemporary (own observation). The giraffe spends a considerable proportion of the day walking (e.g. 5 hours according to [21]) and travels roughly 3 to 5 km per day on average [4, 34]. | | | | | |
| ***canter***  The locomotion is a three-beat movement with a moment of suspension; the tail is usually curled up, sometimes over the back, while cantering. This swifter of the two gaits of the giraffe is only done on rather short distances and for short periods of time [2]. In most cases, canter is an escape movement (e.g. from predators, vehicles or conspecifics), but can also be used as a targeted movement (e.g. towards a conspecific, apparently threatening; own observation). In canter, the giraffe can travel with 56 km per hour [26]. Small obstacles can be jumped over in canter or in a similar movement [2]. The giraffe cannot maintain the canter movement for more than several minutes due to increasing blood pressure [9]. | | | | | |
| ***graze***  The giraffe assumes a position as when *drinking* from a ground level water source (spreading legs, and often also bending carpal joints) to reach the ground. Literature states that the giraffe hardly ever makes use of feeing from the herbaceous layer (summarised in [26]). In own observations though giraffes were seen to spend considerable time grazing [35]. During grazing, a giraffe can change the position of one of its forelegs to enhance its range [35]. Grazing must not be confused with *geophagy*. | | | 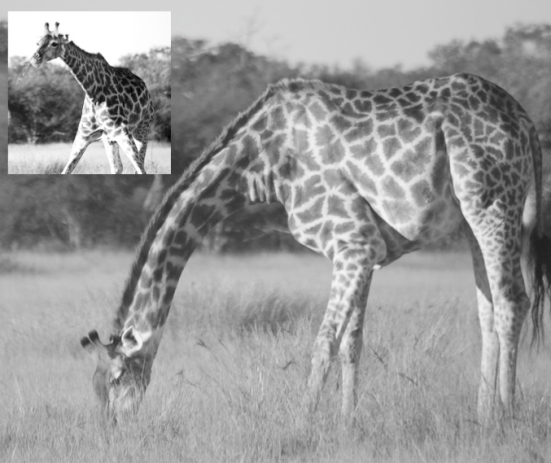 | | |
| ***geophagy***  The giraffe assumes the typical drinking position to reach down to ground-level where it licks soil or even bites pieces of it off to ingest it. Often followed by chewing and/or tongue movement / licking (own observation). Geophagy seems to be more common in regions of poor mineral supply [36] and was seen frequently during own observations in Hwange National Park, where the sandy Kalahari soil is known to contain a very limited amount of micronutrients [54]. | |  | | | |
| ***osteophagy***  The animal bends down to the ground and picks up bone material to chew on it, eventually dropping the piece again [37, 38]. Osteophagia has been reported in captive animals more often than in free-ranging ones and is assumed to be a result of poor supply of dietary minerals [39, 40] . | | | | | |
| ***scavenge***  Parts of a carcass other than bones are chewed on, or ingested [40]. Giraffe in captivity and in the wild were observed to scavenge on carcasses of different herbivore species, including giraffes [26, 40]. Comparably to osteophagy and geophagy, scavenging in the giraffe is presumably an indicator of nutritional stress, as e.g. lacking minerals [30]. Giraffe cows have also been reported to eat the afterbirth [41]. | | | | | |
| ***rest (lying)***  The trunk touches the ground with the giraffe lying in sternal position, legs are tucked in or folded up under the body. The head is carried with an erect or slightly bent neck. When lying down, front legs are bent first, followed the hind legs [23]. Mostly giraffe calves are seen to lie down, but animals of all age classes do so [42]. In adults it is only done for rather short periods of time, presumably to *sleep*, with the head resting on the body [43]. | |  | | | |
| ***drowse***  The animal stands with a rather relaxed neck and lowered head and seems not to *scan*. Rumination can occur while drowsing [23]. Mostly, ears are not carried attentively but are relaxed as well (own observation). These resting phases are rather short and sometimes follow rumination; most apparent during the heat of the day [23]. | | | | | |
| ***yawn***  The animal opens its mouth and sometimes the tongue is protruded, the lower jaw might move sideways (own observation). | | | | | |
| ***sleep***  Lying on the ground in a sternal position (as when resting), neck curled, head resting on the animal‘s hip or thigh and eyes closed [43]. The body position is quite obvious, but the physiological state of sleeping can only be assumed [comp. 62]. As in other ungulate species, juvenile giraffes seem to spend more time sleeping then adult ones [43, 62]. | | | | | |
| ***rub (object)***  The giraffe rubs its body / neck / head against an object. Rubbing on objects is presumably done to get rid of an itch, as the giraffe‘s rather unstable balance does not allow to scratch an itch with its own legs, as seen in other ungulates. Giraffes can also walk and stand over shrubs that are somewhat higher than animal’s trunk to rub their bellies on it by moving back and forth (own observation). | |  | | | |
| ***lick object***  The giraffe licks the surface of an object other than soil or other mineral donators, also followed by nibbling on it. Licking an object seems to be more frequent in juvenile than in adult giraffes (own observation). The tongue is presumably used to investigate on the texture of an object [23]. Not to confuse with the abnormal repetitive behaviour of object licking in captive animals. | | | | | |
| ***paw***  The animal stomps the ground with one foreleg, once or repeatedly. Pawing is rather rare and seems to be a displacement activity caused by an external irritation which is considered as a possible threat by the giraffe [23]. Pawing is sometimes followed by or precedent to *snorting*, which also seems to be a reaction to an irritation; it is mostly to be seen as an interruption of *scanning* and a presumed function of pawing might be considered to be an alarm signal as well [comp. 63]. | | | | | |
| ***snort***  The giraffe blows air out through widened nostrils by a short expulsive and clearly audible grunt. In most cases, the animal is standing and scanning one particular spot or direction; snorting is interpreted in the giraffe as a reaction to a potentially threatening irritation, as it is observed in other ungulates as well [9; comp. 63 respectively]. It might function as a warning signal for conspecifics; in own observations, dominant giraffe bulls were seen and heard to snort immediately after the displacement of an inferior bull. | | | | | |
| ***self-grooming***  The animal licks or bites its own body or legs. Biting or licking the own body is assumed to provide a relief from an itch or another unpleasant local sensation; only several parts of the body can be reached with the animal’s own mouth, in other cases *rubbing* is presumably the method of choice (own observation). | | | | | |
| ***buck***  The animal jumps while cantering or even more or less on the spot, contemporary lowering its neck. Bucking in adult animals is not to confuse with playful jumping in calves, though it resembles the movement. It seems to be a defence reaction against birds such as oxpeckers and might in some cases be followed by *cantering* for a short distance (own observation). Bucking is often preceded by shaking of head and neck to get rid of birds [9]. Calves might buck for the same reason at times. | | | | | |
| ***tail swishing***  The giraffe uses its tail to brush off flies almost on its entire trunk [9]. Tail swishing might also be a sign of irritation in alert situation. | | | | | |
| ***predator defence***  The giraffe lifts its front legs quickly (either successively or both at a time), kicking or stomping them hard on the ground or eventually on a specific target [2, 9]; kicking is also done with the hind legs [26]. Giraffes have been observed to kill lions with these foreleg stomps [2, 9]. This defence behaviour is never directed towards conspecifics [9]. In young animals, a quite similar behaviour can be seen but is done in a playful context with other giraffe calves and is often combined with running or even bucking (own observation). | | | | | |
| ***idle rumination***  A young animal or calf performs a chewing motion as seen in ruminating animals, but no bulge is seen moving down the throat and returning between the bouts; seen in calves under the age of 6 months [42]. | | | | | |
