# Supplementary material for: Behavioural inventory of the giraffe (Giraffa camelopardalis)
Source: BMC Res Notes. 2012 Nov 22;5:650. doi: 10.1186/1756-0500-5-650 (PMC3599642; doi:10.1186/1756-0500-5-650)
Supplement: Additional file 2: Table S2 — Abnormal repetitive behaviours [16,20,26,30,45,46]. [file 1756-0500-5-650-S2.doc]

**Table 2** **Abnormal repetitive behaviours**

| ***tongue playing*** | A persistent twisting movement of the tongue outside the animal’s mouth; not licking an object, and not during or shortly after *feeding*, *geophagy*, *browsing* [26, 45]. |  |
| --- | --- | --- |
| ***object licking*** | The animal uses its tongue on an object that is neither food nor a mineral donator, repeatedly and persistently over a lengthy period of time [16, 30]. |  |
| ***mane biting*** | Biting or chewing the mane of a conspecific for more than some seconds, repeatedly, and not in a grooming context [20, 46]. |  |
| ***vacuum chewing*** | The animal repeatedly performs a chewing motion without prior food intake, and when not ruminating [20]. |  |
| ***pacing*** | The animal walks a definite short path, repeatedly and without a discernible purpose [16]. Pacing is an abnormal repetitive behaviour which has been described in many different captive wildlife species, including the giraffe [16]. However, in giraffe oral abnormal repetitive behaviours seem to appear more often then locomotory ones. |  |
