# Supplementary material for: Behavioural inventory of the giraffe (Giraffa camelopardalis)
Source: BMC Res Notes. 2012 Nov 22;5:650. doi: 10.1186/1756-0500-5-650 (PMC3599642; doi:10.1186/1756-0500-5-650)
Supplement: Additional file 3: Table S3 — General Interactions [5,18,19,23,27,42,44,47-50],[59]. [file 1756-0500-5-650-S3.doc]

**Table 3 General Interactions**

| ***approach*** | One animal moves towards a conspecific, obviously not in order to threaten, but seeking proximity. Approaching appears considerably frequent when bulls join groups and attempt to investigate each individual; these approaches might be followed by *investigation* or *nuzzling* (own observation). |
| --- | --- |
| ***nuzzling*** | A tactile encounter with conspecific by animal‘s nose or muzzle to conspecific‘s nose or any other area then flanks or anogenital area. This greeting ritual seems to be more frequent between cows and calves then among adult giraffes. Pratt and Anderson [42] refer to it as *nasofrontal greeting*, Bashaw et al. [47] as *muzzle/muzzle*. Nuzzling seems to strengthen the social bonds between animals, as it is seen very often in cows, directed to calves before suckling [42.] |
| ***follow*** | One animal stays in proximity to another one and follows its movements in walk. In own observations, this distance was found to be from one body length up to about 60 metres. Following is common in bulls, sometimes prior to the establishment of a mate guarding courtship, or when cows do not approve of the bull’s affiliation. Also, calves have a tendency follow each other [42]. Mature cows were described to follow the movement of their calves [27]. |
| ***yield*** | The giraffe gets out of the way of another giraffe or interrupts approach to change its own way, avoiding the other individual. Yielding is often to be seen in subdominant bulls that get chased away by other bulls (see *displace*), but also cows sometimes yield from approaching bulls; Bashaw [18] refers to yielding as ‘avoiding’. |
| ***displace*** | One animal drives a conspecific off by an agonistic action, which can be an approach in walk or *canter*, or merely standing and staring. The dominance gestures in giraffe are rather subtle to the observer, but seem to be perceived by conspecifics over a considerable distance (Compare *dominance gesture*). Pratt and Anderson [27] report a distance of 40-80 meters between two bulls showing obvious gestures of dominance and submission. In own observation, a distance of more than 100 meters was estimated in similar situations. Displacing is an enhanced form of the *dominance gesture*, aiming to drive the opponent off. In Bashaw [18], dominance gestures are summarised as *non-contact aggression*. |

| ***necking / rubbing (social)*** | One giraffe rubs its head or neck against a conspecific‘s body, sometimes leading to an entwining of the necks. Rubbing (sometimes referred to as necking) seems to be more of a social purpose then to scratch an itch and is frequently seen in young bulls, sometimes leading to *sparring* [19, 48]. | |  | | | |
| --- | --- | --- | --- | --- | --- | --- |
| ***bump*** | One giraffe pushes another one with its chest. Bumping into another giraffe is common in calves, cows and most frequently seen in young bulls [19, 27]. In adult and subadult bulls it is not to confuse with a *mounting attempt*, where the bull investigates another giraffe first and then also pushes it with its chest [19]. Bumping sometimes appears to be an invitation for further social behaviour, such as *sparring* or *necking* [5]. | | | | | |
| ***grooming*** | One animal grooms another one’s body or crest by licking or biting. This is most common in mothers grooming their calves, and appears rather rarely in adult bulls [49]. Not to confuse with the licking of a cow‘s anogenital area or flanks by a bull, which is a part of *investigating.* | | | | | |
| ***mount*** | One animal stands right behind or on the side of another one, lifting its front legs on to conspecific‘s body, attempting to mount it. In adult bulls, the mounting attempt is usually preceded by pushing the other animal with the chest and lower neck [50]. In calves, it is rather a sudden jump onto the other animal, usually in play with another calf [5]. Mounting attempts seem to be very common among premature bulls; the mounted animal does sometimes not tolerate, but in other cases even ignores being mounted and continues feeding [23]. |  | | | | |
| ***nursing attempt*** | One animal attempts to suckle on a cow‘s udders. The unsuccessful nursing attempts are mostly seen in calves, which approach a cow that is already nursing another calf [27]. Sometimes subadult or adult animals also approach the nursing act, and try to suckle themselves; cows are reported not to allow any other calve then their own to nurse [27]. | | | | | |
| ***sparring*** | The giraffe swings its head against the body of the sparring partner with comparably soft blows. The two, or up to eight animals can stand parallel or antiparallel or in a different angle to each other [48]. The motion of sparring resembles that of a fight, but sparring is considerably slower and less vigorous; sparring seems to be a social behaviour, exaggerated by bulls, and, rarely, even by cows [5]. Sparring develops slowly, sometimes initiated by *necking* and can continue over hours [48]. It is usually interrupted oftentimes for several minutes to scan, or even to ruminate. In literature, only cows in captivity are mentioned to spar, usually against a bull [48]. During our own observations in HNP, only one young but mature cow was observed to join a sparring match between young bulls. | | | |  | |
| ***flehmen*** | The giraffe raises its head up, sometimes with the nose line tilted above the horizontal, and curls up the upper lip, inhaling deeply. The flehmen response is frequently performed by bulls after *urine testing* in the process of *investigating*, showing long saliva threads hanging from the mouth [44]. By inhaling deeply, the giraffe presumably uses its Jacobson‘s organ to assess the cow‘s cyclic state [44, 57]. | | |  | | |
|  |  | | |  | | |
| ***Investigate*** | The animal licks or sniffs a conspecific‘s anogenital area or flanks. Investigating is most common in bulls directed towards adult and subadult cows. By investigating the bull intends to stimulate the cow to urinate, and subsequently might perform *urine testing* [19]. The behaviour can be conducted by bulls or cows, and in both cases directed to either sex (own observation). | | |
| ***vocalise*** | The giraffe produces infrasound vocalizations, sometimes by performing a ‘head throw’ behaviour (von Muggenthaler, Baes, Hill, Fulk, Lee, unpublished results). Infrasound in the giraffe is hypothesised to be a means of long- and short-distance communication. In captivity, infrasound vocalization has been reported in adult bulls and cows (von Muggenthaler, Baes, Hill, Fulk, Lee, unpublished results). | | | | | |
|  |  | | | | |  |
| ***lick urine*** | The giraffe licks another giraffe‘s urine from the ground. Licking urine from the ground is not to confuse with *urine testing*, which is performed by adult and sometimes by subadult bulls after stimulating a cow to urinate. Licking urine in bulls is sometimes followed by a *flehmen* response [44]. Adult cows were also seen to lick urine of other giraffes from the ground (own observation). Classified as an  *interaction* due to its presumed character of communication. | | | | | |
