# Supplementary material for: Behavioural inventory of the giraffe (Giraffa camelopardalis)
Source: BMC Res Notes. 2012 Nov 22;5:650. doi: 10.1186/1756-0500-5-650 (PMC3599642; doi:10.1186/1756-0500-5-650)
Supplement: Additional file 4: Table S4 — Bull Cow Behaviour [19,23,27]. [file 1756-0500-5-650-S4.doc]

**Table 4 Bull Cow Behaviour**

|  |  |  |
| --- | --- | --- |
| ***mate guard*** | The bull directs one cow away from conspecifics, or intends to prohibit approach of other bulls. Bulls might keep proximity of less than one animal’s body length, in other cases, the bull remains in a distance of up to 30 metres; the intensity of mate guarding and the proximity a bull keeps to the guarded cow seems to depend on presence of other bulls, and presumably on the cow’s oestrus state [19]. |
|  |  |  |
| ***urine testing*** | The bull tests cows urine (often when the cow urinates), usually leading to a *flehmen* response. Urine testing is a part of the investigation procedure; by nuzzling a cow’s flanks the bull stimulates the cow to urinate [19]. As she urinates, the bull catches some urine in its mouth; as suggested by Innis [23] the bull might be able to assess the cow‘s reproductive state by urine testing. |
|  |  | |
| ***laufschlag*** | The bull stands close behind a cow or follows a moving cow and lifts one front leg (repeatedly) to touch the cows hind leg or flank. It can be assumed that laufschlag is a preliminary investigation of the cow‘s readiness to mate; during our own observations this behaviour was only seen when the bull was *mate guarding*. | |
|  |  | |
| ***mating*** | The bull rocks its body weight on the hind quarters and lifts front legs to mount the cow. The bull’s penis is erect, subsequent intromission and several vigorous pelvic thrusts [19, 23]. The cow does not necessarily stand still during the copulation [19]. Ejaculation follows immediately to intromission, as within 2 s [19]. Since cows hardly stand still, mating is mostly preceded by several mating attempts [19, 27]. | |
