# Supplementary material for: Behavioural inventory of the giraffe (Giraffa camelopardalis)
Source: BMC Res Notes. 2012 Nov 22;5:650. doi: 10.1186/1756-0500-5-650 (PMC3599642; doi:10.1186/1756-0500-5-650)
Supplement: Additional file 5: Table S5 — Bull-Bull Behaviour [9,18,23,41,60]. [file 1756-0500-5-650-S5.doc]

**Table 5** Bull-Bull Behaviour

|  |  |
| --- | --- |
| ***dominance gesture*** | According to Drews’ definition [64] - any type of behaviour in an agonistic encounter between two individuals characterised by a consistent hierarchy outcome in favour of one of them, and default yielding response of the opponent rather then escalation. Often an erect posture while standing still or a tensed and arched neck when moving, by which a bull intends to express its social rank, or tries to challenge another individual. The visual signs of dominance differ remarkably and can only be interpreted by the social context (also see *displace* and *submission*; [5, 9]). A dominance gesture can only be assessed by its social context and, in some cases, might be somewhat arbitrary. In other cases dominance is made very clear to the counterpart animal and obvious to the observer (see Discussion). |
|  |  |
| ***submission*** | Any type of behaviour shown by a subordinate bull in an agonistic encounter in order to avoid escalation [64]; most obvious is *yielding*, that subsequently results in an avoidance of or retraction from a social ranking dispute [5]. Like *dominance*, submission is expressed in various ways and can only be interpreted from the social context. |
|  |  |
| ***fight*** | One bull attacks by swinging head and neck against his opponent‘s body and legs with very powerful and fast blows [5, 23]. To increase their stability, the fighting individuals spread their front and hind legs and lean against each other with their hindquarters touching [23]. Sometimes the attack with the head is preceded by a bump with the rear, as seeking to push against the opponent and lean against it at a time. It is also referred to as *contact aggression* [18]. |

| ***play fight*** | The movement resembles that of a *fight*. The blows with head and neck are delivered quite vigorous, but not as powerful and as fast as in a fight. As in a fight, the participants often lean against each other with their hindquarters to increase stability; a play fight is usually also interrupted at times for the participants to scan, as if taking a break and after several minutes go back to the play fight, which does not occur in a serious fight (own observation). |  |
| --- | --- | --- |
