# Supplementary material for: Behavioural inventory of the giraffe (Giraffa camelopardalis)
Source: BMC Res Notes. 2012 Nov 22;5:650. doi: 10.1186/1756-0500-5-650 (PMC3599642; doi:10.1186/1756-0500-5-650)
Supplement: Additional file 6: Table S6 — Cow - Bull Behaviour [23,27]. [file 1756-0500-5-650-S6.doc]

**Table 6** Cow - Bull Behaviour

|  |  |  |
| --- | --- | --- |
| ***escape from bull*** | A cow escapes from a bull’s affiliation attempt (*mate guard*, or *investigation*). Usually in canter, the cow intends to withdraw from a bull’s affiliation; persistent bulls keep up with the cow and restrict her from approaching conspecifics of both sexes; the cow’s canter appears somewhat slower than when escaping from predators or vehicles (own observation). |
|  |  |  |
| ***tolerate affiliation*** | The cow tolerates the bull‘s investigation, or mounting attempts. The cow tolerates the bull‘s investigation and mounting (attempts) by standing still and not *walk* or *canter* away from the bull; the cow can even ignore the investigating bull and keep browsing [23]. |
|  |  | |
| ***stimulated urination*** | The cow urinates subsequently due to stimulation by a bull’s investigation. When bulls *investigate* cows, the cows often react by urinating, leading to *urine testing*; sometimes cows are stimulated to urinate merely by a big bull walking past them [27]. | |
|  |  | |
| ***tolerate mating attempt*** | The cow tolerates bull‘s mounting attempt, eventually leading to copulation. Not necessarily standing still, but not obviously trying to escape from the bull’s close-up [own observation, 27]. Cows which are just about to come into oestrus might tolerate the bull‘s investigation but not the mounting attempt; a cow might keep browsing while the bull mounts her, or she keeps walking, but not *escaping* from the bull [27]. | |
