# Supplementary material for: Behavioural inventory of the giraffe (Giraffa camelopardalis)
Source: BMC Res Notes. 2012 Nov 22;5:650. doi: 10.1186/1756-0500-5-650 (PMC3599642; doi:10.1186/1756-0500-5-650)
Supplement: Additional file 7: Table S7 — Behavioural Interactions by Calves [5,42,47]. [file 1756-0500-5-650-S7.doc]

**Table 7** **Behavioural Interactions by Calves**

|  |  |
| --- | --- |
| ***tail-chew*** | The calf chews on another individual‘s tail. In most cases, the calf chews its mother‘s tail, sometimes on the tail of another juvenile [49]. |
|  |  |
| ***suckle*** | Juvenile animal sucks milk from cow‘s udders. It was reported that the nursing relationship in giraffe is exclusively limited to one cow and her calf by Pratt and Anderson [42]. They also suggest that nursing serves as a strengthening of the mother-calf bond, and not only for nutrition. the suckling act can be initiated by either the calf of the mother [42]. |
|  |  |
| ***play*** | A variety of behavioural patterns in the immature giraffe can be interpreted as play behaviour. This includes running, bucking, kicking with fore- or hind legs, and biting other individuals; play behaviour appears most commonly among young calves, as they play with each other, perform play running and jumping solitarily, or try to play with adult giraffes [5]. This behaviour excludes *play fighting.* |
