# Supplementary material for: Behavioural inventory of the giraffe (Giraffa camelopardalis)
Source: BMC Res Notes. 2012 Nov 22;5:650. doi: 10.1186/1756-0500-5-650 (PMC3599642; doi:10.1186/1756-0500-5-650)
Supplement: Additional file 8: Table S8 — Maternal behaviour [41,42,55]. [file 1756-0500-5-650-S8.doc]

**Table 8** **Maternal behaviour**

|  |  |
| --- | --- |
| ***neonate cleaning*** | The cow licks the new-born calf dry, eventually eating the foetal membranes [52,59]. |
|  |  |
| ***suckling initiation*** | The cow approaches her calf and stares at it; once the calf realises it approaches the udders and begins to nurse [42]. |
|  |  |
| ***suckling prevention*** | The cow does not allow the calf to suckle, by simply moving away or moving one of her hind legs forward (own observation, [42]). No other calves but the own one is permitted to suckle [42]. |
|  |  |
| ***nurse*** | The mother stands still and lets the calf suckle, while she abstains from *browsing* but only *scans*. [42,59]. The suckling act is mostly terminated by the cow, not by the calf [42]. |
|  |  |
| ***olfactory bonding*** | The mother touches the young calf on head, mane, back or rump with her nose, and licks the calf’s body; the sniffing appears most frequently on neonates, but also with calves of several days of age, probably to strengthen the mother-calf bond [42,59]. |
